# Sex and Age-specific Association between Isokinetic Knee Extensor Muscle Strength and Diabetes Mellitus: A Cross-sectional Study from the Sports Program Service Study

**DOI:** 10.31662/jmaj.2025-0111

**Published:** 2025-07-02

**Authors:** Takahisa Ohta, Junzo Nagashima, Hiroyuki Sasai, Kazushige Sasaki, Naokata Ishii

**Affiliations:** 1 Research Team for Promoting Independence and Mental Health, Tokyo Metropolitan Institute for Geriatrics and Gerontology, Tokyo, Japan; 2 Yokohama Sports Medical Center, Yokohama, Japan; 3 Division of Cardiology, Department of Internal Medicine, St. Marianna University School of Medicine, Kawasaki, Japan; 4 Department of Life Sciences, Graduate School of Arts and Sciences, The University of Tokyo, Tokyo, Japan

**Keywords:** epidemiology, logistic regression, cross-sectional study, isokinetic knee extensor muscle strength, diabetes mellitus

## Abstract

**Introduction:**

Diabetes mellitus (DM) is an increasing public health concern in Japan and worldwide, highlighting the need for effective prevention. Lower limb muscle strength, a modifiable factor, may influence glycemic control, potentially in sex- and age-specific ways. This study examined the association between isokinetic knee extensor strength and DM prevalence in a large Japanese cohort.

**Methods:**

A total of 14,017 Japanese individuals (men: 6,227; women: 7,790) aged 18-89 years participated in this study. All participants completed the maximum voluntary isokinetic knee extensor strength test (60°/s) and medical examination. DM was defined as blood glucose ≥126 mg/dL, hemoglobin A1c ≥6.5%, or hypoglycemic medication. Multivariable odds ratios (ORs) and 95% confidence intervals were calculated using the lowest quartile knee muscle strength category as a reference after adjusting for potential confounders.

**Results:**

The mean age of the participants was 50.1 years (standard deviation; 15.3); altogether, 1,225 participants (8.7%) had DM. Multivariable ORs of the highest quartile muscle strength were 0.61 (0.48-0.78) for men and 0.65 (0.57-0.88) for women, using the lowest quartile muscle strength as a reference. Knee extensor muscle strength was inversely associated with DM (p for trend <0.001 and 0.003, men and women, respectively). The inverse association was consistently observed in men aged 40 years and older, whereas it was less apparent in older women.

**Conclusions:**

Isokinetic knee extensor muscle strength is inversely associated with the prevalence of DM, with potential differences by sex and age. These findings may help inform DM risk assessment and prevention strategies.

## Introduction

Diabetes mellitus (DM), a significant risk factor for cardiovascular disease, represents a critical public health concern worldwide, contributing to increased mortality ^(1)^. With DM currently affecting more than 422 million individuals globally, projections estimate that Japan will see its DM prevalence exceed 8.3% by 2030 ^(2)^. In Japan alone, the financial burden of DM care has already surpassed $11 million ^(3)^, with expectations of further increases due to the complications associated with the disease, such as renal disorders, which elevate medical costs and the need for polypharmacy ^(4)^. Consequently, the development of strategies to prevent or delay the onset of DM is of paramount importance. Prior research has identified several modifiable risk factors for DM, including smoking, alcohol intake, obesity, cardiorespiratory fitness, and grip strength, alongside non-modifiable factors such as age and genetics. Notably, muscle strength emerges as a cost-effective preventive measure, underscored by previous studies ^(5)^.

Skeletal muscle, the largest organ involved in energy metabolism within the human body, is crucial for thermogenesis ^(6)^. Epidemiological evidence has highlighted a bidirectional link between sarcopenia―a common muscle condition in older adults―and DM, underscoring the importance of both muscle mass and strength in predicting health outcomes, with muscle strength being a superior indicator ^(7)^. Resistance exercise, known to augment muscle strength, has been shown to be beneficial in glycemic control ^(8)^, thereby establishing muscle strength as a significant predictor of DM, all-cause mortality, and cardiovascular diseases ^(5), (9)^.

Grip strength, a simple, practical, and economical indicator of overall muscle strength, particularly in the upper limbs ^(10)^, contrasts with the less frequently used assessment of lower limb muscle strength owing to its higher cost and measurement complexity. Nevertheless, lower limb strength offers better predictions of falls, physical fitness, and performance metrics such as walking speed and stair-climbing ability, and is associated with physical activity levels ^(11)^. Exploring the relationship between lower limb muscle strength and DM could thus propel advancements in DM preventative medicine. Despite this, recent systematic reviews and meta-analyses have not addressed lower extremity muscle strength ^(5)^.

Emerging research has begun to explore the link between lower extremity muscle strength and DM, with studies by Suwa et al. ^(12)^ and Wong et al. ^(13)^ indicating an inverse relationship between lower limb muscle strength and DM prevalence, a connection not observed with grip strength alone. Moreover, superior lower limb muscle function, as shown in activities such as the chair-stand test, has been suggested to have a preventive effect on DM progression ^(14)^. Although the chair-stand test can provide a simple and valid indicator of lower limb muscle function, its performance depends not only on muscle strength but also on balance and endurance ^(13)^. Moreover, the chair-stand test requires the coordination of multiple limbs, joints, and muscle groups ^(15)^. Meanwhile, the increasing interest in safely but directly quantifying local muscle strength through isokinetic dynamometry in clinical and sports rehabilitation underscores its potential value ^(16)^. Therefore, direct assessment of knee extensor muscle strength could illuminate the underlying mechanisms linking muscle strength with DM. In addition, investigating the precise association between lower limb muscle strength, as measured by isokinetic contraction, and DM is crucial. On the basis of the previously mentioned evidence, we hypothesize that isokinetic knee extensor strength may play a preventive role in reducing the risk of DM. Moreover, potential differences in this association by sex and age have not been fully explored and may reflect underlying hormonal, physiological, or behavioral variations ^(17)^.

This focus not only enriches discussions around the neurological dimensions but also complements existing insights into skeletal muscle mass and quality. With the anticipated increase in DM prevalence in Japan, understanding these specific associations becomes increasingly vital ^(2)^. Thus, this study aims to elucidate whether the association between isokinetic knee extensor muscle strength and DM prevalence differs by sex and age group, using large-scale clinical data from Japanese adults, providing a more comprehensive perspective on prevention strategies in the public health context.

## Materials and Methods

### Study population and design

This investigation focused on a cohort of Japanese adults, ranging in age from 18 to 89 years, who participated in the Sports Program Service (SPS) ^(18)^, an initiative launched by the government of Yokohama City in 1998 and aimed at enhancing citizen health through the Yokohama Sports Medical Center. The SPS was publicized through government websites and leaflets, inviting voluntary participation. A tiered participation fee structure was implemented, requiring participants younger than 65 years to contribute ¥15,000 (approximately $103), whereas those 65 years old and older were charged a reduced fee of ¥7,500 ($52). Participants were asked to complete a self-administered questionnaire that gathered information on lifestyle factors, including smoking and drinking habits and any medications taken for DM. Moreover, they underwent medical examinations and physical fitness assessments. From April 1998 to July 2019, a total of 18,161 individuals successfully completed the SPS program. Subsequently, participants were excluded if they were younger than 18 years, lacked complete muscle strength data, or had incomplete blood sampling information.

Informed consent was obtained from all participants, permitting their participation in the SPS and the use of their data for this study. This research was conducted in strict adherence to the ethical standards laid out in the Declaration of Helsinki and the Strengthening the Reporting of Observational Studies in Epidemiology statement ^(19)^, receiving ethical clearance from the Research Ethics Committee of the Yokohama Sports Medical Center (approval number K-2019-007).

### Clinical examination

All participants underwent a medical examination. Height and weight were measured without shoes on a height-weight scale (WB-510, Tanita Co., Tokyo, Japan), and body mass index (BMI) was calculated (kg/m^2^). The resting blood pressure was measured using the Riva-Rocci-Korotkov method (mercury sphygmomanometer). Blood samples were collected after 12 hours of fasting and blood glucose, glycated hemoglobin, total cholesterol, high-density lipoprotein cholesterol (HDL-C), low-density lipoprotein cholesterol (LDL-C), and triglyceride (TG) levels were analyzed using a chemistry analyzer (Roche INTEGRA 400 plus, Roche International Ltd., Basel, Switzerland).

### Isokinetic knee extensor muscle strength

The knee extensor muscle strength was measured twice using an isokinetic dynamometer (Cybex Humac Norm 770; Computer Sports Medicine, Inc., Stoughton, MA, USA). Trained medical staff instructed the participants to sit at the knee and ankle joint angles of 90° and to warm up. Subsequently, the participants performed an isokinetic maximum knee extension at 60°/s. The measurement was performed twice with an interval of a couple of minutes. The maximum value was used as the knee extensor muscle strength (Nm) and adjusted for body weight (Nm/kg).

### Assessment of diabetes

DM was defined on the basis of the criteria outlined by the Japan Diabetes Society, which include the following: fasting blood glucose level ≥126 mg/dL, hemoglobin A1c (National Glycohemoglobin Standardization Program) ≥6.5%, self-reported physician-diagnosed DM, and/or the use of glucose-decreasing medication ^(20)^.

### Assessment of other covariates

Information on alcohol consumption habits, smoking habits, and medications for DM was collected using a self-reported questionnaire.

### Statistical analysis

All the statistical analyses were performed using SPSS version 25 (IBM Corp., Armonk, NY, USA). To reduce potential bias, multiple imputations were performed to create 20 datasets. The auxiliary variables that accounted for the missing data were all covariates.

Considering age and sex differences in DM, we created decade-based categories for each sex (18-29, 30-39, 40-49, 50-59, 60-69, and ≥70). In each age-stratified category, participants were divided into quartiles based on knee extensor muscle strength (lowest, lower, higher, and highest). The quartiles were combined to create an age-adjusted group. Continuous and categorical variables were expressed as median (interquartile range) or mean (standard deviation) and percentage, respectively.

Logistic regression analysis was performed to clarify the association between each explanatory variable and the prevalence of DM. Moreover, multivariate analysis was performed to investigate the association between knee extensor muscle strength and the prevalence of DM after adjusting for confounding factors such as sex, age, smoking habits, drinking habits, systolic blood pressure, LDL-C, HDL-C, TGs, and BMI. Multivariable odds ratios (ORs) and 95% confidence intervals (CIs) were calculated using the lowest knee extensor muscle strength as a reference. Sex-stratified analysis was also conducted. In addition, to examine potential sex differences in the association between muscle strength and DM, a multivariable analysis including an interaction term between muscle strength and sex was conducted. Stratified analyses by sex and age group (18-39, 40-64, and ≥65 years) were also conducted, and an interaction term between sex and muscle strength was included in the model. Statistical significance was set at p < 0.05.

## Results

**Figure 1.**
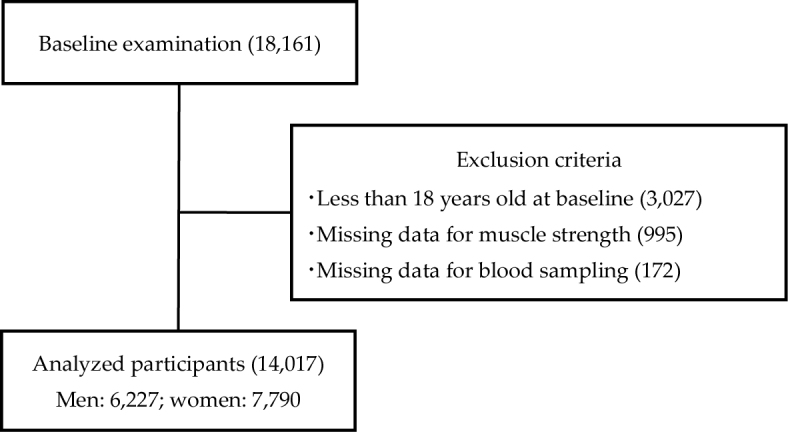
Flow of participant selection.

During 1998-2019, 18,161 individuals participated in the SPS. Of them, by exclusion criteria, 14,017 participants (men: 6,227; women: 7,790; mean age 50.1 [range 18-89] years) were included in the analysis (Figure 1). Overall, 1,225 participants (8.7%, self-reported, including those under pharmacologic treatment: n = 722; biochemically determined DM: n = 503) had DM (Table 1). BMI was inversely correlated with muscle strength.

**Table 1.** Demographic, and Clinical Characteristics of Participants (18-89 years) Classified by Isokinetic Knee Extensor Muscle Strength.

Variables			All N = 14,017 (6,227/7,790)	Knee extensor muscle strength			
				Q1 (lowest) n = 3,498 (1,555/1,943)	Q2 (lower) n = 3,506 (1,557/1,949)	Q3 (higher) n = 3,510 (1,559/1,951)	Q4 (highest) n = 3,503 (1,556/1,947)
Number of cases (men/women)			1,225 (720/505)	529 (318/211)	306 (179/127)	236 (131/105)	154 (92/62)
KNES	Men	N・m/kg	2.38 (0.55)	1.86 (0.38)	2.27 (0.35)	2.50 (0.35)	2.97 (0.40)
	Women		1.73 (0.41)	1.35 (0.28)	1.64 (0.27)	1.81 (0.26)	2.19 (0.31)
Age		year	50.1 (15.3)	50.7 (15.4)	50.2 (15.3)	50.0 (15.2)	49.6 (15.2)
Height		cm	162.3 (8.8)	161.7 (8.9)	162.3 (8.8)	162.6 (8.8)	162.8 (8.7)
Weight		kg	60.2 (11.8)	63.7 (13.6)	60.7 (11.4)	59.2 (10.7)	57.0 (10.0)
BMI		kg/m^2^	22.7 (3.5)	24.3 (4.2)	23.0 (3.3)	22.3 (3.0)	21.4 (2.6)
Blood glucose		mg/dL	100.5 (17.0)	102.0 (21.3)	100.9 (17.6)	99.9 (14.8)	99.2 (13.0)
Hemoglobin A1c		%	5.2 (0.6)	5.6 (0.7)	5.2 (0.5)	5.1 (0.5)	5.1 (0.4)
Total cholesterol		mg/dL	210.7 (37.3)	213.1 (37.9)	210.5 (37.5)	210.0 (36.2)	209.0 (37.3)
HDL cholesterol		mg/dL	63.3 (16.6)	60.8 (16.3)	62.3 (16.3)	63.5 (16.5)	66.8 (16.9)
LDL cholesterol		mg/dL	120.8 (32.3)	127.0 (33.0)	121.8 (32.1)	119.9 (31.2)	114.5 (31.5)
Triglycerides		mg/dL	98.0 (70.9)	112.2 (75.4)	102.5 (87.8)	94.2 (59.7)	83.1 (51.9)
SBP		mmHg	117.5 (16.9)	120.5 (17.6)	118.1 (16.9)	116.4 (16.7)	115.0 (16.0)
Smoking		n (%)	1,728 (12.3)	550 (16.0)	462 (13.2)	380 (10.8)	336 (9.6)
Drinking		n (%)	6,642 (47.7)	1,554 (44.5)	1,652 (47.2)	1,695 (48.3)	1,741 (49.9)

Data were shown as mean (standard deviation) or frequency (percentage). BMI: body mass index; HDL: high-density lipoprotein; KNES: knee extensor muscle strength; LDL: low-density lipoprotein; Q: quartile; SBP: systolic blood pressure.

Table 2 shows the association between the prevalence of DM and each confounding variable. The prevalence of DM was lower in women than in men (p < 0.001). In the BMI category, especially in the ≥25 kg/m^2^, showing ORs (95% CIs) 2.89 (2.20, 3.80, p < 0.001). Moreover, an inverse linear association was observed between knee extensor muscle strength and the prevalence of DM (p < 0.001).

**Table 2.** Associations between Demographic, Clinical, and Physical Variables and Diabetes Mellitus: Univariable Analysis.

Potential risk factors	No. of participants	No. of cases	Prevalence rate*	Odds ratios (95% CI)	p-Value
Sex					
Men	6,227	720	115.6	1.00 (reference)	-
Women	7,790	505	64.8	0.53 (0.47-0.60)	<0.001
Age, year					
18-39	3,930	87	22.1	1.00 (reference)	-
40-64	6,883	616	89.5	4.34 (3.46-5.45)	<0.001
≥65	3,204	522	162.9	8.60 (6.82-10.85)	<0.001
Current smoking					
None	12,211	1059	86.7	1.00 (reference)	-
Yes	1,728	160	92.6	1.08 (0.90-1.28)	0.419
Current drinking					
None	7,288	642	88.1	1.00 (reference)	-
Yes	6,642	578	87.0	0.99 (0.88-1.11)	0.824
Body mass index, kg/m^2^					
<18.5	1,122	64	57.0	1.00 (reference)	-
18.5 to <25.0	9,851	703	71.4	1.27 (0.97-1.66)	0.081
≥25.0	3,044	458	150.5	2.89 (2.20-3.80)	<0.001
Knee extensor muscle strength					
Lowest	3,513	422	120.1	1.00 (reference)	<0.001
Lower	3,500	325	92.9	0.75 (0.64-0.87)	
Higher	3,512	269	76.6	0.61 (0.52-0.71)	
Highest	3,492	209	59.9	0.47 (0.39-0.55)	

CI: confidence interval. *Prevalence rate is per 1,000 persons.

**Figure 2.**
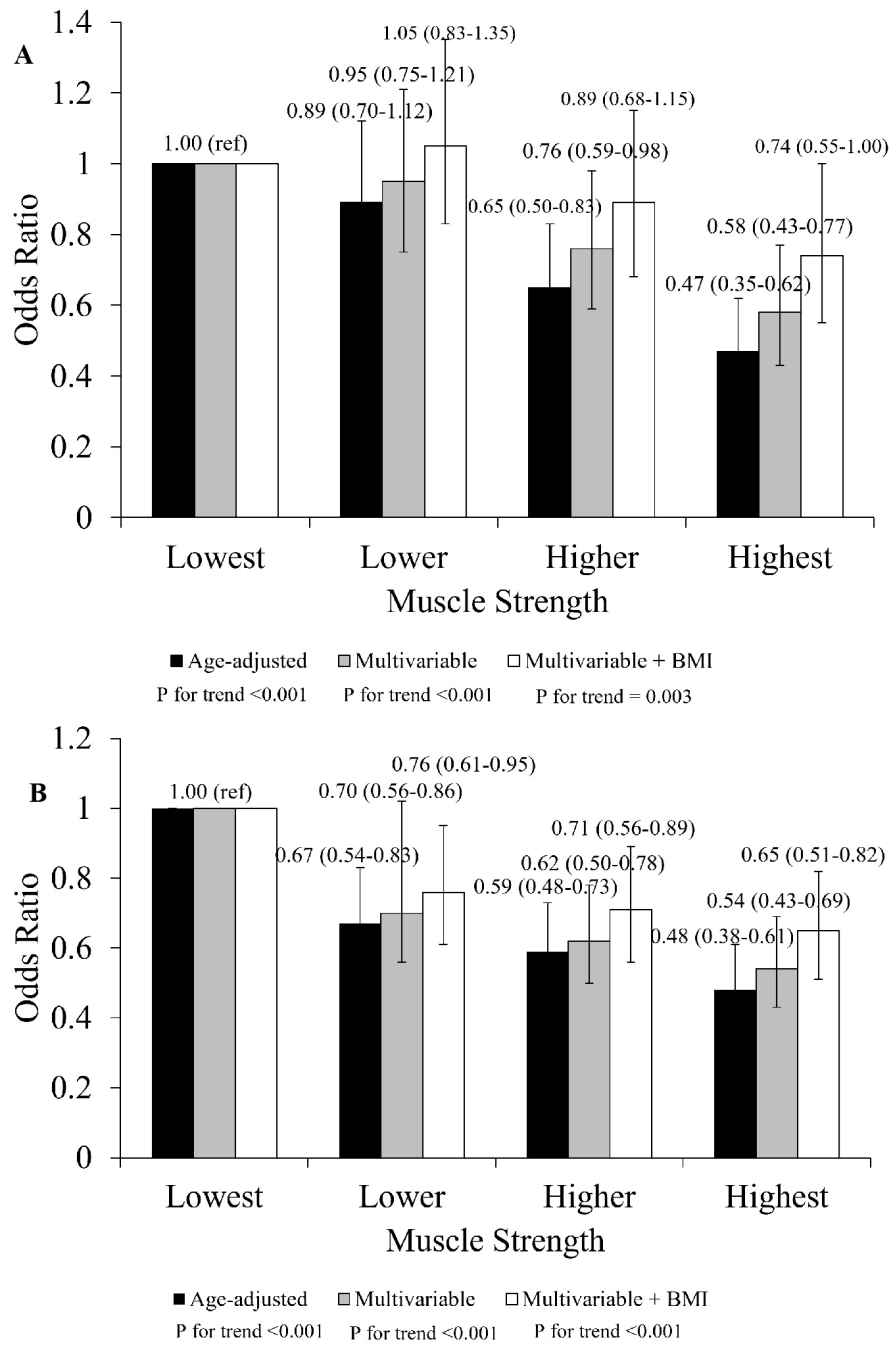
Sex-stratified multivariable logistic regression analysis: adjusted odds ratios for diabetes mellitus based on isokinetic knee extensor strength and BMI in Japanese adults. A: men, B: women; BMI: body mass index. Adjusted for age, high-density lipoprotein cholesterol, low-density lipoprotein cholesterol, triglycerides, systolic blood pressure, current smoking habits, and current drinking habits.

An inverse association between the prevalence of DM and knee extensor muscle strength persisted after adjusting for potential confounders for men (p < 0.001) and women (p < 0.001) (Figure 2). Furthermore, in multivariate analysis with BMI added as a covariate, higher muscle strength was associated with lower ORs for DM prevalence (p for trend < 0.001 for men, p for trend = 0.003 for women). An interaction between sex and muscle strength did not observe (p for interaction = 0.909).

In age- and sex-stratified analyses, an inverse association between knee extensor strength and DM prevalence was observed in both men and women aged 40-64 years (men: OR = 0.58, 95% CI: 0.40, 0.85; women: OR = 0.52, 95% CI: 0.33, 0.82; both p for trend < 0.001) (Table 3). A similar trend was also observed in men aged ≥65 years (OR = 0.55, 95% CI: 0.37, 0.82; p for trend = 0.003), whereas no clear pattern was observed in women of the same age group. While no significant interaction between sex and muscle strength was observed in the 18–39 age group (p = 0.758), significant interactions were found in both the 40–64 and ≥65 age groups (both p < 0.001).

**Table 3.** Sex-Stratified Multivariable Logistic Regression Analysis: Adjusted Odds Ratios for Diabetes Mellitus Based on Isokinetic Knee Extensor Strength according to Age Category (18-39, 40-64, and ≥65).

			Knee extensor muscle strength					
Age category			Q1 (lowest)	Q2 (lower)	Q3 (higher)	Q4 (highest)	p for trend	p for interaction
18-39	Men	Case (%)	24 (4.9)	8 (1.6)	7 (1.4)	15 (3.0)		0.758
		AOR* (95% CI)	1.00 (reference)	0.48 (0.20-1.12)	0.56 (0.22-1.38)	1.52 (0.70-3.30)	0.759	
	Women	Case (%)	11 (2.2)	12 (2.4)	5 (1.0)	5 (1.0)		
		AOR* (95% CI)	1.00 (reference)	2.35 (0.92-5.97)	1.34 (0.41-4.36)	1.07 (0.49-5.67)	0.975	
40-64	Men	Case (%)	150 (22.4)	70 (10.4)	71 (10.6)	59 (8.8)		<0.001
		AOR* (95% CI)	1.00 (reference)	0.49 (0.36-0.67)	0.59 (0.43-0.83)	0.58 (0.40-0.85)	<0.001	
	Women	Case (%)	117 (11.1)	69 (6.6)	50 (4.8)	30 (2.9)		
		AOR* (95% CI)	1.00 (reference)	0.74 (0.53-1.03)	0.67 (0.47-0.99)	0.52 (0.33-0.82)	<0.001	
≥65	Men	Case (%)	97 (24.6)	83 (21.1)	81 (20.6)	55 (14.0)		<0.001
		AOR* (95% CI)	1.00 (reference)	0.82 (0.58-1.15)	0.81 (0.57-1.15)	0.55 (0.37-0.82)	0.003	
	Women	Case (%)	61 (15.0)	54 (13.3)	50 (12.3)	41 (10.1)		
		AOR* (95% CI)	1.00 (reference)	0.94 (0.62-1.42)	0.99 (0.65-1.51)	0.86 (0.54-1.38)	0.622	

AOR: adjusted odds ratio; CI: confidence interval; Q: quartile. *Adjusted for age, high-density lipoprotein cholesterol, low-density lipoprotein cholesterol, triglycerides, systolic blood pressure, current smoking status, current drinking status, and body mass index.

## Discussion

This study investigated the association between isokinetic knee extensor muscle strength and the prevalence of DM in a large sample of Japanese adults. We found that higher knee extensor muscle strength was associated with a lower prevalence of DM, independent of potential confounders. Moreover, stratified analyses revealed that the association differed by sex and age group, being stronger in men and less apparent in older women. These findings suggest that lower limb muscle strength, particularly as measured by isokinetic testing, may be a useful indicator of DM risk, with potential variation across demographic subgroups.

This study revealed that knee extensor muscle strength could serve as a viable indicator for the prevalence of DM, which differs from handgrip strength assessments. Traditionally, handgrip strength has been a favored metric for evaluating muscle fitness owing to its convenience, low cost, and prognostic value ^(5), (21)^. However, this method may not fully capture age-related decreases in muscle strength, and it may be less sensitive to the changes in muscle mass and strength after repeated bouts of resistance exercise, particularly those targeting larger muscle groups in the lower extremities ^(22), (23)^. Epidemiological research has further indicated a lack of association between handgrip strength and DM risk ^(24)^, with findings showing a pronounced reduction in lower extremity muscle strength but not in upper extremity strength among individuals with DM ^(25)^. Hence, the outcomes of this study advocate for the inclusion of muscle strength evaluations in clinical settings that have access to lower extremity isokinetic muscle strength measurement apparatus, moving beyond sole reliance on grip strength assessments. In addition, evidence suggests that not only back muscle strength but also the strength of upper and lower limbs is linked to the incidence of DM ^(26), (27)^. These observations align with our findings, reinforcing the premise that knee extensor muscle strength could be used as an indicator of whole-body muscle strength and thus a more robust predictor of DM than grip strength, despite the relatively few studies on this topic.

The association between muscle strength and DM has been extensively documented, with particular attention to the role of lower extremity muscle function in recent studies ^(5)^. Unlike grip strength, which can be straightforwardly assessed, evaluating the strength of lower extremities is generally more complex owing to the involvement of various factors, including balance and the coordination of multiple limbs, joints, and muscle groups. Isokinetic muscle strength measurement offers a method to indirectly assess muscle quality by providing a more focused evaluation of muscle strength devoid of these confounding factors. From the perspective of muscle quality, the force-generating capacity is partly dependent on the proportion of type II fibers within the muscle. These fibers are capable of fast and powerful contractions, differing significantly from type I fibers, which have lower contractile velocity but higher oxidative capacity to maintain force production. Populations exhibiting higher isokinetic muscle strength had reduced prevalence of DM. This correlation may be attributable to the enhanced glucose uptake capabilities of type II fibers relative to type I fibers. Type II muscle fibers, given their glycolytic nature, are more efficient in glucose uptake, which could explain the lower incidence of DM in individuals with greater isokinetic strength, highlighting the metabolic benefits of having a higher proportion of these fibers ^(28)^.

A critical insight from this study is that the association between muscle strength and DM persists even after adjusting for BMI in both sexes. Given the well-documented role of obesity as a significant risk factor for DM ^(29)^, recent understandings have positioned DM within the spectrum of inflammatory diseases that often originate from obesity-related conditions. The process of adipocyte hypertrophy, stimulated by overnutrition, leads to an escalated release of inflammatory cytokines. This increase in cytokine levels enhances inflammation within adipose tissues, thereby contributing to the development of insulin resistance ^(30)^. Moreover, obesity has been implicated in upregulating the expression of inflammatory macrophages within human skeletal muscle, further establishing a connection among inflammation, insulin resistance, and the onset of DM. The findings of this study underscore the significance of muscle strength as a vital consideration in formulating interventions aimed at the prevention or management of DM, especially in individuals with obesity. Our findings suggest that enhancing lower limb strength may reduce DM risk independently of obesity, highlighting its potential role in prevention strategies. This underscores the potential of muscle-strengthening exercises as a component of comprehensive strategies to combat DM, highlighting the importance of focusing on muscle health in addition to managing weight and addressing inflammatory processes in obesity. This view is further substantiated by large-scale prospective cohort studies showing that regular engagement in muscle-strengthening activities is independently associated with a lower risk of type 2 diabetes, thereby reinforcing the role of muscular fitness as a key target in diabetes prevention ^(31)^.

Although no overall interaction between sex and muscle strength was observed, age-stratified analyses showed that the inverse association with DM was consistently observed in men but less evident in older women. This may suggest that the strength-DM relationship weakens with age in women, possibly owing to hormonal changes after menopause, age-related decreases in muscle quality, or lifestyle and metabolic differences ^(32)^. Epidemiological studies have also reported that the prevalence of type 2 diabetes is generally higher in men than in women, although this gap narrows with age, particularly after menopause ^(17)^. Such background differences may partly explain the variation observed across sex and age groups.

Given the cross-sectional nature of this study, establishing a causal link between knee extensor muscle strength and DM remains elusive. One plausible interpretation is that DM itself may precipitate a decrease in muscle strength. Previous research documented a significant reduction―up to 50%―in knee extensor muscle strength over a span of three years among individuals diagnosed with DM ^(33)^. This substantial decrease in muscle strength could potentially be attributed to the development of insulin resistance, which has been shown to impair muscle functionality by inhibiting the activity of Akt kinase. Akt kinase plays a crucial role in regulating the mammalian target of rapamycin pathway ^(34)^, essential for muscle growth and maintenance. Furthermore, insulin resistance is associated with increased levels of pro-inflammatory cytokines, such as interleukin-6 and tumor necrosis factor-alpha, which contribute to diminished muscle function ^(34), (35), (36)^. Moreover, chronic hyperglycemia has also been shown to promote skeletal muscle atrophy by inducing oxidative stress and protein catabolism ^(37)^. Conversely, it is also conceivable that reduced muscle strength could lead to the development of DM. Skeletal muscle, being a primary site for glucose utilization, benefits from muscle-strengthening activities such as resistance exercise through the upregulation of glucose transporter type 4 ^(38)^. In addition, regular muscle-strengthening exercises aid in maintaining or enhancing skeletal muscle mass and strength, thereby improving glycemic control and insulin sensitivity ^(39)^. Consequently, the World Health Organization advocates engaging in muscle-strengthening activities at least two days per week ^(40)^. These contrasting mechanisms may synergistically produce the intricate relationship between muscle strength and DM, highlighting the bidirectional nature of their association. Future longitudinal studies are imperative to disentangle the causal dynamics underlying the observed correlations, paving the way for more effective prevention and management strategies for DM.

This study’s methods, characterized by the large-scale and direct measurement of muscle strength using isokinetic testing, represent a significant advantage. Unlike community-based physical fitness assessments such as the chair-stand test, which incorporate various factors beyond mere muscle strength but offer simplicity and the capacity to examine numerous individuals, the rigorous approach of isokinetic muscle strength testing in this research provides a robust and representative dataset from a substantial participant cohort. Nonetheless, the present study is not without its limitations. Firstly, the cross-sectional design precludes the determination of causality between muscle strength and DM. This gap necessitates further longitudinal studies to elucidate the dynamics of their relationship. Moreover, the potential impact of DM on muscle weakness, possibly through mechanisms such as diabetic neuropathy, remains unexplored owing to the unavailability of data regarding diabetic complications. Secondly, the absence of dietary information and inflammatory markers, such as C-reactive protein, introduces the possibility of residual bias, given these factors are known to influence the onset and progression of DM. Thirdly, although prediabetes is also a clinically relevant condition, we focused our analysis on DM to ensure a clear and consistent outcome definition. Given the distinct pathophysiological features of prediabetes, including early insulin resistance and β-cell dysfunction, future studies should explore its relationship with muscle strength in more detail ^(41)^. Lastly, this study did not account for the habitual physical activity levels of participants, despite the established dose-response relationship between physical activity and a reduced risk of DM incidence ^(42)^. Addressing these limitations in future research could enhance our understanding of the complex interactions between muscle strength and DM, potentially leading to more targeted prevention and management strategies for this chronic condition.

In conclusion, this study revealed that elevated isokinetic knee extensor muscle strength is inversely associated with the prevalence of DM. Interestingly, the association between isokinetic muscle strength and DM was independent of BMI, indicating that vigorous physical activity (i.e., resistance exercise training), focused on muscle strength development, is crucial in managing DM. The findings underscore the significance of regularly engaging in muscle-strengthening activities not only as a strategy for preventing DM but also for averting the loss of physical fitness. However, although this cross-sectional study lays the groundwork for understanding the association between muscle strength and DM, it also underscores the need for further investigation. Specifically, longitudinal research is needed to explore the long-term benefits of directly quantifying lower limb strength across diverse demographics. Such future studies could provide more definitive evidence on the role of muscle strength in preventing and managing DM, potentially informing more targeted and effective intervention strategies.

## Article Information

### Conflicts of Interest

None

### Sources of Funding

This research was financially supported in part by an internal operational grant from the Yokohama Sports Medical Center.

### Acknowledgement

We thank the participants and staff members at the Yokohama Sports Medical Center. We thank Editage (www.editage.com) for the English language editing. We wish to express our profound gratitude to the late Professor Naokata Ishii. His discerning intellect and deep expertise were essential to the conception and refinement of this manuscript. From its earliest stages, Professor Ishii provided thoughtful guidance and scholarly advice that shaped the core of the work. His dedication to the advancement of sports and exercise medicine, as well as his unwavering support for this study, shall be remembered with the utmost respect and appreciation.

### Author Contributions

Takahisa Ohta and Hiroyuki Sasai designed the study; Takahisa Ohta and Junzo Nagashima collected the data; Takahisa Ohta analyzed the data; Takahisa Ohta wrote the first draft; Junzo Nagashima, Hiroyuki Sasai, Kazushige Sasaki, and Naokata Ishii oversaw the entire project. All authors reviewed and contributed to the critical revisions of the manuscript and approved the ﬁnal version of the manuscript.

### Approval by Institutional Review Board (IRB)

Research Ethics Committee of the Yokohama Sports Medical Center (approval number K-2019-007).

Naokata Ishii deceased.
